# Social isolation produces no effect on ultrasonic vocalization production in adult female CBA/CaJ mice

**DOI:** 10.1371/journal.pone.0213068

**Published:** 2019-03-05

**Authors:** Laurel A. Screven, Micheal L. Dent

**Affiliations:** Department of Psychology, University at Buffalo, SUNY, Buffalo, NY, United States of America; University of Missouri Columbia, UNITED STATES

## Abstract

Mice produce ultrasonic vocalizations (USVs) in a wide variety of social contexts, including courtship, investigation, and territorial defense. Despite the belief that mouse USVs are innate, social experience may be necessary for mice to learn the appropriate situation to emit USVs. Mouse USVs have been divided into categories based on their spectrotemporal parameters, but it is currently unclear if social experience changes these parameters (e.g., frequency and duration) or the proportion of calls from each category produced. Social isolation has been found to influence USV production in male mice. To investigate the influence of social isolation on vocal behavior in female mice, recordings were made of USVs emitted to unfamiliar male and female mice by subjects with one of three types of social experience. Twenty-four adult female CBA/CaJ mice either lived alone, lived with other females only, or lived with other females and had limited access to a male. Mice were recorded while in isolation, ensuring all recorded USVs were from the female of interest. Vocalizations were separated into nine categories and peak frequency, duration, and bandwidth were measured for every call. Socially isolated mice did not produce significantly more USVs or USV types than socially experienced mice. Social isolation did not have a significant effect on the features of USVs, suggesting production of USVs may not be learned in female mice.

## Introduction

Mice (*Mus musculus*) emit complex ultrasonic vocalizations (USVs) during social encounters with conspecifics, leading researchers to assume their role as a communication signal (e.g. [1]). USVs are believed to function as a means of social recognition and courtship, to elicit approach behavior, as well as to communicate aggression [2–5]. USVs vary across spectrotemporal parameters, including frequency, duration, and intensity. These features are utilized to parse USVs into categories by researchers (e.g. [1, 6–8]). Mouse USVs have become a commonly used model for human communication, making it critical to understand more about USV production and the function of these rodent vocalizations.

A fundamental area of research on USV concerns when and under what conditions mice produce vocalizations. Studies on USV production have focused almost entirely on male mice, due to the outdated and incorrect assumption that only males vocalize during courtship interactions (e.g., [9]). As a result, USVs recorded from male-female pairs are assumed to originate from the male mouse. Recently, however, several experiments have shown that female mice do vocalize during male-female interactions [10–11]. For example, Neunuebel and colleagues [10] discovered, using microphone arrays, that female mice emit almost 20% of the USVs recorded in male-female pairs.

Socialization is critical for the development of communication signals in many animals. In zebra finches (*Taeniopygia guttata*), young male birds must learn their characteristic songs through tutoring by an older male zebra finch within a sensitive period between 35 and 65 days old [12–15]. Social interaction has also been shown to be important for language acquisition in humans (*Homo sapiens*) through studies of second language learning in infants (reviewed in [16]). As mice are used as models for human communication, it is important to determine the effect of social experience on vocal behavior in mice. In support of the mouse model of human communication, it has also been suggested that there is a learned aspect of USV production in mice (e.g., [17–18]), with mice falling onto a continuum of vocal learning (see [19] for review). Chabout and colleagues [18] found that FoxP2, a gene critical for language production in vocal-learning species, also has a significant effect on USVs in mice. Male heterozygous FoxP2 knockout mice showed decreased bout length to both female urine and anesthetized female mice, suggesting there is, at least in part, a similar genetic mechanism controlling acoustic communication in mice and humans.

The effects of social experience on USV production are a relatively unexplored topic in mice. Although several lines of evidence suggest USV production is innate [7–8], there is data that shows deafening adult male mice changes features of vocalizations (e.g., pitch and noisiness of USVs) [20], indicating that mice require auditory feedback to preserve the spectrotemporal parameters of USVs. It is still unknown if mice need social experience to learn when and in what social contexts in which they produce USVs (see [17–18]). Currently, the only experiment that has investigated the role of chronic social isolation on vocal behavior in mice was conducted by Keesom and colleagues [21]. The authors recorded USVs from pairs of socially isolated or experienced male CBA/J mice. Isolated male mice produced significantly more USVs than socially experienced mice. The greatest number of USVs were emitted when a socially experienced mouse was paired with a socially isolated mouse. Socially housed mice recorded together produced the fewest number of USVs, and isolated mice recorded together emitted an intermediate number of USVs. These findings illustrate that social experience has an effect on the vocal behavior of mice. The results of this experiment are limited due to their exclusive use of male subjects, as well as their inability to determine which mouse of the dyad was producing the recorded USVs. In the present experiment, we recorded from mice individually to determine if socially isolated female mice showed patterns of increased vocal behavior, similar to the socially isolated male mice in the Keesom et al. [21] study. If so, it could indicate that mice potentially learn some aspects of USV production as a result of social experience.

The increased vocal output of Keesom and colleagues’ [21] chronically isolated male mice could also be the result of decreased social competence. Social competence refers to the ability of an animal to make the appropriate behavioral response to an environmental stimulus, often requiring plasticity to reflect the internal state of the animal [22–23]. Plasticity of behavioral responses is believed to induce both short- and long-term phenotypic changes [24] and likely functions to allow animals to change their behavior in response to differences in motivation. The effects of social experience on behavior have been investigated in humans [25], mice [26], hamsters (*Mesocricetus auratus*) [27], zebra finches [28–29], guppies (*Poecilia reticulate*) [30], and cichlids (*Neolamprologus pulcher*) [31]. For example, rhesus monkeys (*Macaca mulatta*) who were isolated from peers during development showed social incompetence through increased submissive, agonistic, and avoidance behaviors compared to monkeys who had been reared with same-age peers [32]. This suggests that early social experience with peers is critical for developing the behavioral repertoire necessary for social interaction with conspecifics in adulthood. Additionally, Keesom et al. [21] reported that isolated male mice showed decreased social competence by displaying inappropriate behaviors, such as mounting, towards other male mice. Their results imply that social experience with conspecifics is necessary for mice to develop social competence, and that social isolation likely impairs behavioral plasticity to changing environmental contexts and internal states.

An important topic that must be addressed is how social experience affects USV production in female mice. The majority of the existing research on the effects of social experience on vocalization production has focused on male mice and how prior experience with female mice affects calling behavior (e.g., [33–34]). The goal of this study was to examine how vocal behavior differs between chronically isolated female mice and female mice who have experience with either other females or with both male and female mice. Due to the increased numbers of USVs observed in male mice by Keesom and colleagues [21], we hypothesized isolated female mice would vocalize significantly more than socially experienced females. Features of USVs were not hypothesized to differ between the socially housed and individually housed female mice, since strain-specific USVs are thought to be innate (e.g., deaf mice produce normal USVs [8]). In contrast to our hypothesis, no significant differences were found in the production of USVs across female mice with different levels of social experience, with socially isolated mice producing a statistically similar number of USVs as socially experienced mice. The number of USV types produced within a single recording session also did not differ across levels of social experience, with isolated mice producing a similar number of USV types than socially housed mice. Finally, the proportion of USV types emitted did not vary across social exposure conditions. The findings of this experiment illustrate that social experience may play a minor role in the development of vocal behavior in female mice.

## Materials and methods

### Ethics statement

All procedures were approved by the University at Buffalo, SUNY’s Institutional Animal Care and Use Committee [IACUC] under protocol PSY13056N.

### Subjects

Twenty-four female CBA/CaJ mice were used in this experiment. After weaning at 21 days old (P21), females were divided into three groups of eight mice, and all were housed in the same colony room. The first group (isolated) was housed individually for the duration of the experiment. Since the isolated mice were housed in the group colony room, they were not acoustically isolated but had no physical or social contact with other mice for the duration of the experiment. The second group (F Exposed) was housed in two groups of four female mice until the later phases of the experiment, during which they were still only exposed to female mice, but in different housing configurations (see Housing apparatus section). The third group (M/F Exposed) was also housed in two groups of four mice during the first two phases of the experiment, after which they were housed with both a male and a female mouse (see Housing apparatus section). Group housed females were identified by tail markings. Mice in all three groups remained in the same housing condition throughout the duration of the experiment (e.g., isolated mice remained socially isolated for the entire experiment).

Female estrous cycles were monitored using vaginal cytology by examining vaginal wall cells for the presence or absence of leukocytes, cornified epithelial, and nucleated epithelial cells (see [35] for full methodology). Females were only recorded during the diestrus phase of their estrous cycle to keep the phase consistent between subjects and across recording sessions. Diestrus was chosen due to female mice’s tendency to produce more USVs during this phase compared to mice in sexually receptive phases [36]. Estrous cycle tracking was completed within four hours of the recordings.

The colony room was kept in standard conditions (mean temperature 72 ± 3°F). Mice were kept on a reverse day/night cycle (lights off at 6 am and lights on at 6 pm). All recordings were done during the dark portion of their light cycle under red light conditions. Food and water were provided ad libitum. After mice completed all phases of the experiment, they were moved on to other experiments. Mice will be sacrificed at a later time in accordance with AMVA guidelines.

## Housing apparatus

**Fig 1:**
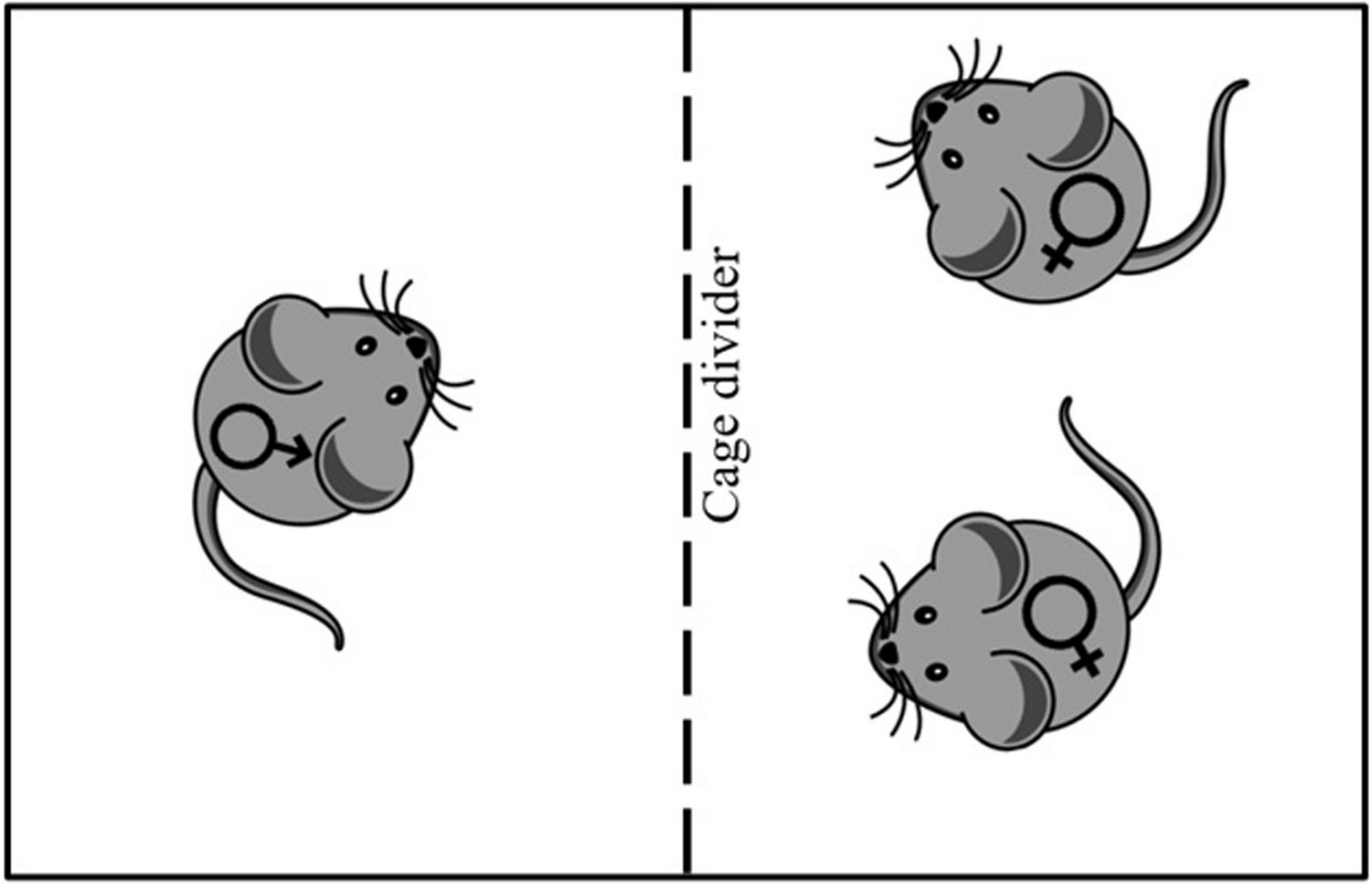
The exposure cage apparatus was a standard mouse cage bisected with chicken wire. Mice in the M/F and F Exposed conditions lived in the exposure cages for four weeks.

Beginning at 13 weeks old, mice in the F Exposed and M/F Exposed groups lived in bisected standard mouse cages (30 x 19 x 13 cm) for four weeks to allow olfactory, acoustic, and limited tactile experience with a female (F Exposed) or a male (M/F Exposed) mouse. Complete physical contact was limited to prevent mating. Cages were bisected with a piece of chicken wire (Fig 1). In each cage, two females were placed on one side of the chicken wire divider and one male or one unfamiliar female was placed on the other side. Two females were placed in the same exposure cage to avoid short-term social isolation as a side effect of the exposure conditions. It is important to note that Isolated mice did not live in bisected cages at any point, although it is unlikely that this housing condition produced any significant negative side effect on mice. Mice in the F Exposed and M/F Exposed conditions did not show any significant changes in calling behavior after living in the bisected cage.

### Recording apparatus

**Fig 2:**
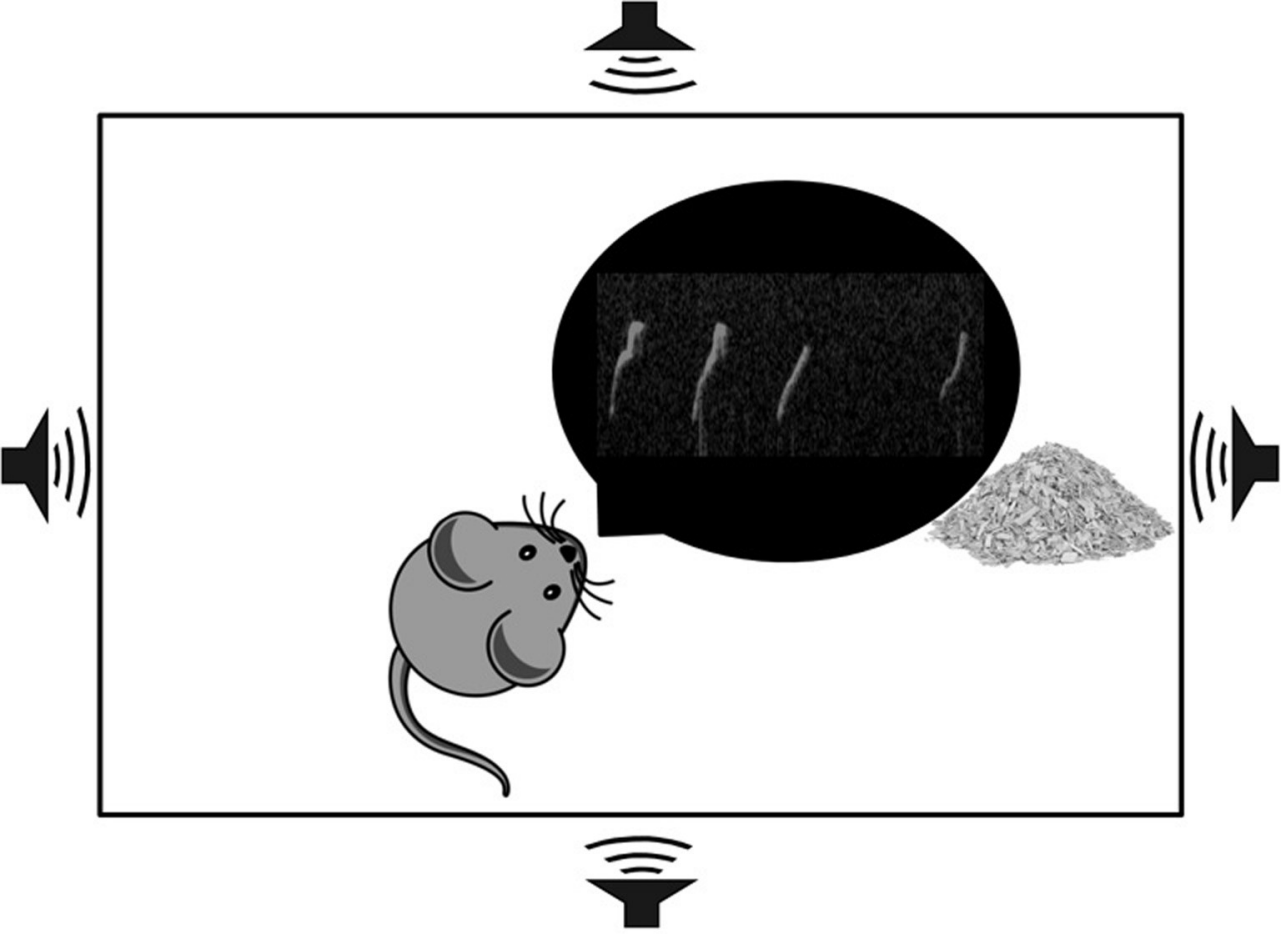
Schematic of the recording apparatus. A standard mouse cage was placed into an anechoic chamber. A condenser microphone was placed on each side of the apparatus, pointing towards the opposite wall. Mice were placed inside the recording apparatus alone for the entire duration of the recording session. Recordings lasted for five minutes following a 15 second habituation to the apparatus.

Recordings took place in a clean, empty home cage (30 x 19 x 13 cm) topped with a slotted metal cover. The cage was placed inside a plastic tub lined with anechoic foam (Sonex sound attenuating foam, 4 cm). A condenser microphone (UltraSoundGate CM16/CMPA, flat frequency response (± 6 dB) between 25 and 140 kHz) was placed on each side of the cage next to an opening covered with chicken wire, pointed towards the center (Fig 2). Stimulus mice were placed in a separate clean home cage, 5 cm from the recording apparatus. Recording mice did not have any physical interaction with the stimulus mice at any point during the experiment, including recording sessions.

### Procedure

#### Recording procedure

It has been shown that what the mouse is doing for the hour before a recording session can have an effect on vocalization production [37]. To standardize the experience of mice before the recording session, all mice were acoustically and socially isolated for one hour before the recording. Following this period of isolation, the female mouse was placed into the recording apparatus and allowed to habituate for 15 seconds. Following habituation, a stimulus mouse (male or female) was placed in a separate home cage 5 cm away from the recording cage. Dirty bedding from the stimulus mouse was placed inside the recording apparatus to ensure the recording mouse could detect the sex of the stimulus mouse. Both the bedding and stimulus mouse were used because it was unclear if either alone would be sufficient in eliciting vocalizations. USVs were recorded onto a laptop computer (Hewlett-Packard Notebook model 15-ac121dx) using Avisoft software and hardware (UltraSoundGate 116). Settings included a 300 kHz sampling rate with a 16-bit format. Because of the directionality of USVs, any USV that came from the stimulus mouse was easily identifiable in the recording due to differences in intensity of the USVs (usually ~30 dB lower in intensity with increased spectral noise than USVs from the subject mouse). Any USV believed to be from the stimulus mouse were not included in the analysis. Recording sessions lasted 5 minutes, after which time the mice were returned to their home cages.

#### Experimental timeline

**Fig 3:**
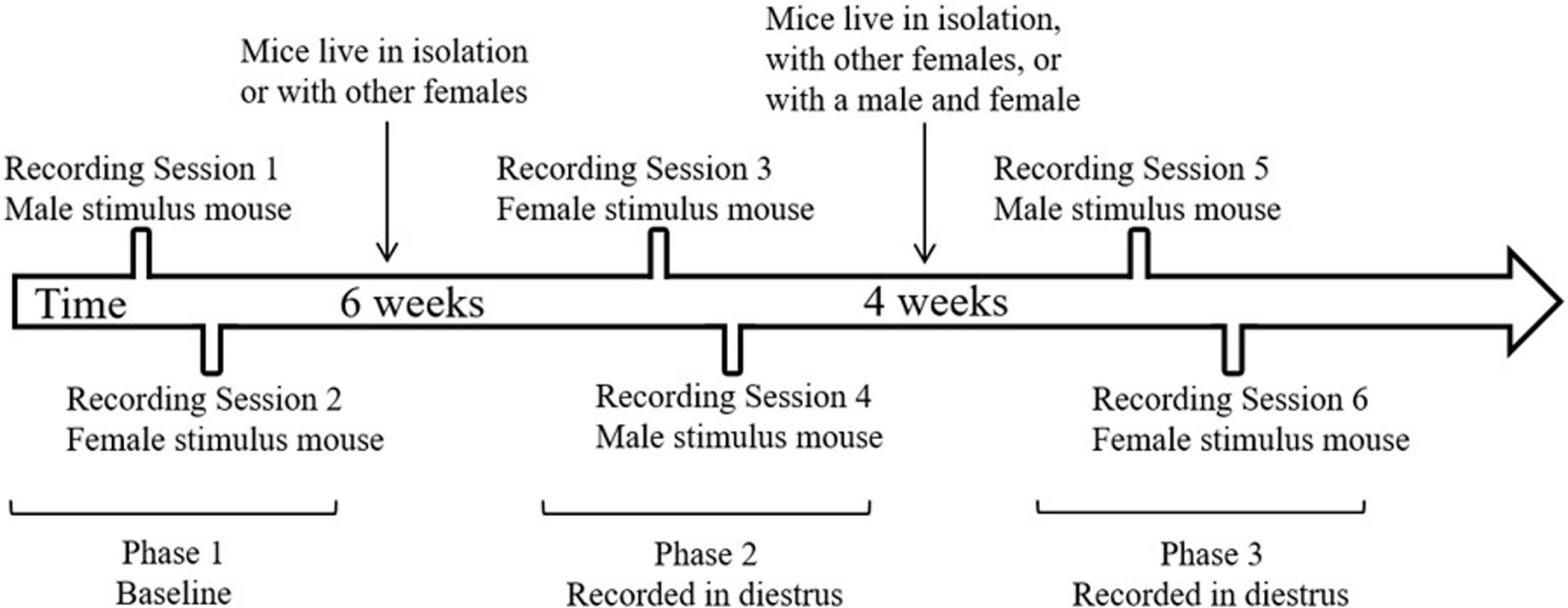
Example timeline of recording sessions for one mouse. The order of the sex of the stimulus mouse was counterbalanced across mice within each phase.

Mice were recorded during phase 1 two times (Fig 3). Each mouse was first recorded on postnatal day 20 (P20) with an unfamiliar, age matched stimulus mouse (e.g., male) and returned to their litter. The following day, on P21, the same mouse was recorded again with another unfamiliar, age matched stimulus mouse of the opposite sex used in phase 1 (e.g., female). Mice were not sexually mature at this age, so recordings in these juvenile mice were not based on estrous cycle. After the completion of phase 1, mice were removed from their litters and placed into one of two housing conditions: isolated or group housed with other females. Mice lived undisturbed in these conditions for six weeks until phase 2 of the recordings began. After this six-week period, estrous cycles of all mice were tracked daily until the mice entered diestrus. Once in diestrus, mice were recorded with an unfamiliar, age matched stimulus mouse (e.g., female) and returned to their home cage. Estrous cycles continued to be tracked daily until the mouse entered diestrus again 4–5 days later. When the mouse entered diestrus, they were recorded with another unfamiliar, age matched stimulus mouse in recording session 3 (e.g., male). After the completion of phase 2, mice were placed into one of three housing conditions: isolated, group housed with females, or group housed with males. Mice lived undisturbed in these housing conditions for four weeks until phase 3 of the recordings began. After this four-week period, estrous cycles were again tracked daily for all mice until the mice entered diestrus. Once in diestrus, mice were recorded with an unfamiliar, age matched stimulus mouse (e.g., male) and returned to their homecage. Estrous cycles continued to be tracked daily until the mouse again entered diestrus 4–5 days later. Mice were recorded for the final time to an unfamiliar, age matched stimulus mouse in recording session 5 (e.g., female). The sex of the stimulus mouse was counterbalanced across mice, so the order in which mice were exposed to a male or female mouse was differed for each subject. Mice lived undisturbed for six weeks between phase 1 and 2 to ensure mice were sexually mature at the time of phase 2. The time between phase 2 and 3 was reduced to 4 weeks to limit the amount of time mice spent in the bisected cages.

### USV analysis

**Fig 4:**
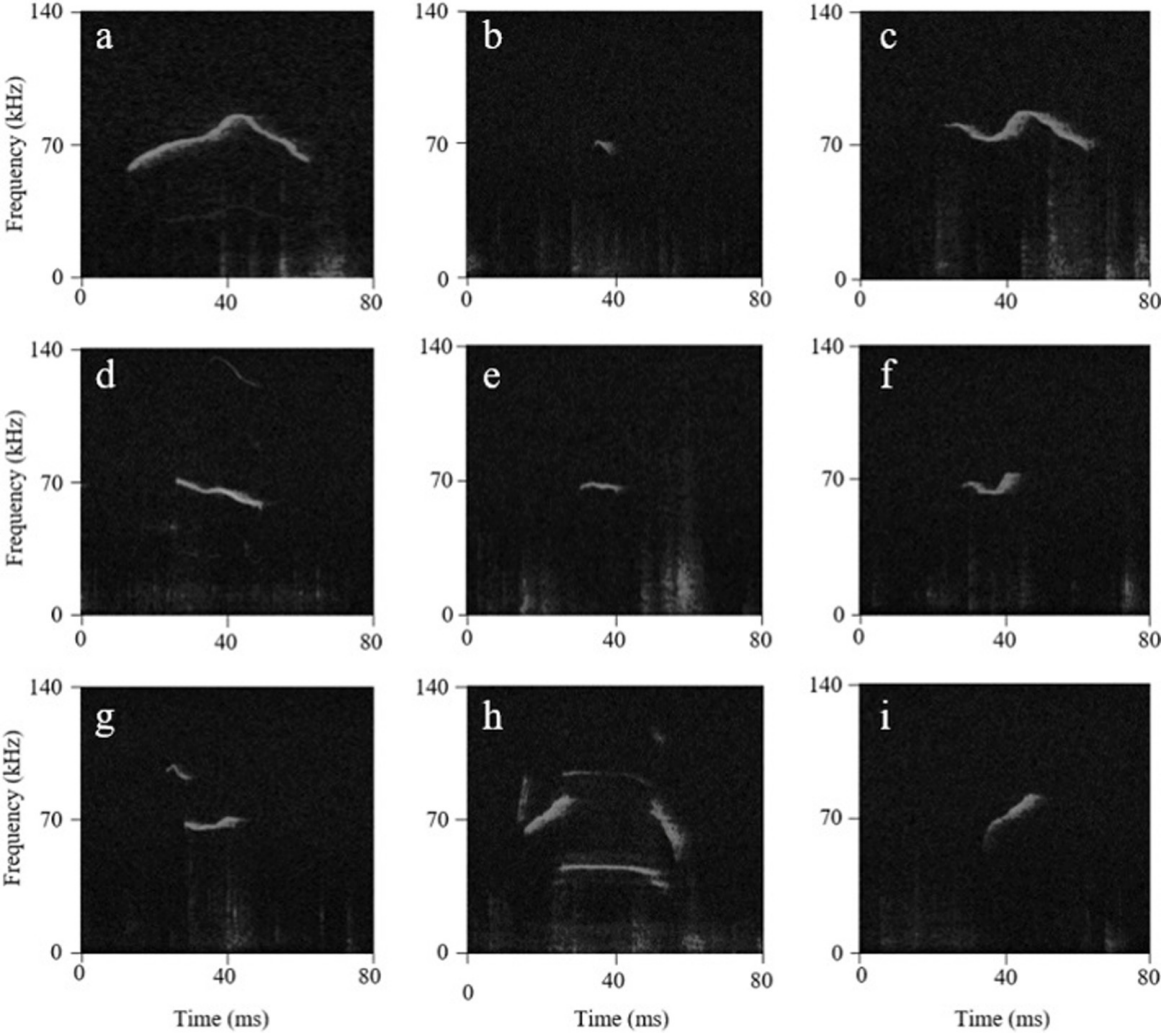
USV categories. Example USV spectrograms from the (a) chevron, (b) chirp, (c) complex, (d) downsweep, (e) flat, (f) inverse chevron, (g) jump, (h) multijump, and (i) upsweep categories.

USV extraction and analyses were conducted using Raven Pro (v. 1.5, Cornell Lab of Ornithology), as used in [37]. Nine USV categories (chevron, chirp, complex, downsweep, flat, inverse chevron, jump, multijump, and upsweep) (Fig 4) previously described [1, 5, 38] were used in this study. A USV was categorized as chevron when it increased in frequency with the highest frequency reaching >5 kHz above the beginning and end frequencies. Chirps were short USVs less than 10 ms in duration. Complex vocalizations were USVs that contained two or more directional changes in frequency and >5 kHz of frequency modulation. Downsweeps were vocalizations that started at a higher frequency than they ended (with the frequency change greater than 5 kHz). Flat vocalizations had less than 5 kHz of frequency modulation. Inverse chevron vocalizations decreased and then increased in frequency with the lowest frequency reaching >5 kHz below the beginning and end frequencies (shaped like a U). Jump vocalizations contained one break in frequency with no break in intensity. Multijump USVs contained at least two jumps in frequency and often contained a harmonic. Upsweeps were USVs increasing in frequency (with the frequency change greater than 5 kHz). Spectrograms were scored by two individuals (not blind by hand and USV parameters required a minimum interrater reliability criterion of 95%. All parameters of calls (peak frequency, duration, and bandwidth) were automatically calculated by Raven Pro. Call category was the only parameter determined by scorers based on the criteria listed above. The numbers of each vocalization type were obtained for each subject for each recording session, and then the peak frequency, duration, and bandwidth were extrapolated for each USV in each category.

### Data analysis

No mouse emitted vocalizations during phase 1, therefore these sessions were not included in the analysis. To analyze the number of USVs emitted within each housing condition, a one-way repeated measures analysis of variance (ANOVA) was conducted for each housing group separately, with recording session as the factor of comparison. No differences were found within housing groups, therefore a Kruskal-Wallis nonparametric test was performed to analyze the mean number of USVs produced in all recording sessions across the three social experience groups, with housing condition as the factor of comparison. A nonparametric test was performed because number of USVs was non-normally distributed across groups. Number of call types produced was also examined within each housing group separately using a one way repeated measures ANOVA, with recording session used as the factor of comparison. Since no differences were found within housing groups, a one-way ANOVA was conducted for the mean number of call types produced across all recording sessions, with housing group as the factor of comparison. Significant main effects were further investigated using Tukey post hoc analyses. Proportions of USVs produced were non-normally distributed, therefore Friedman repeated measures tests were performed to examine differences within each housing group separately for proportions of USVs produced, with recording session used as the factor of comparison. Since no significant differences were found, Kruskal-Wallis tests were used to test for differences in mean proportions of USVs emitted in all recording sessions between mice in the three housing groups. Lastly, features of USVs (duration, peak frequency, and bandwidth) were investigated within each housing condition separately using a one way repeated measures ANOVA, with recording session for each group independently as the factor of comparsion. Significant differences were further investigated using Tukey post hoc tests. Kruskal-Wallis tests were used to examine mean features across all recording sessions between each housing condition. For all analyses, recording sessions in which mice did not emit any USVs were not included. This was done to ensure sessions with no USVs present did not cause false differences in vocal behavior between housing groups. For all analyses that utilized two tests for each parameter (i.e., number of calls, proportion of USVs, and number of USV types emitted), a Bonferroni correction was applied and significant results were denoted by an alpha level less than 0.025.

## Results

**Fig 5:**
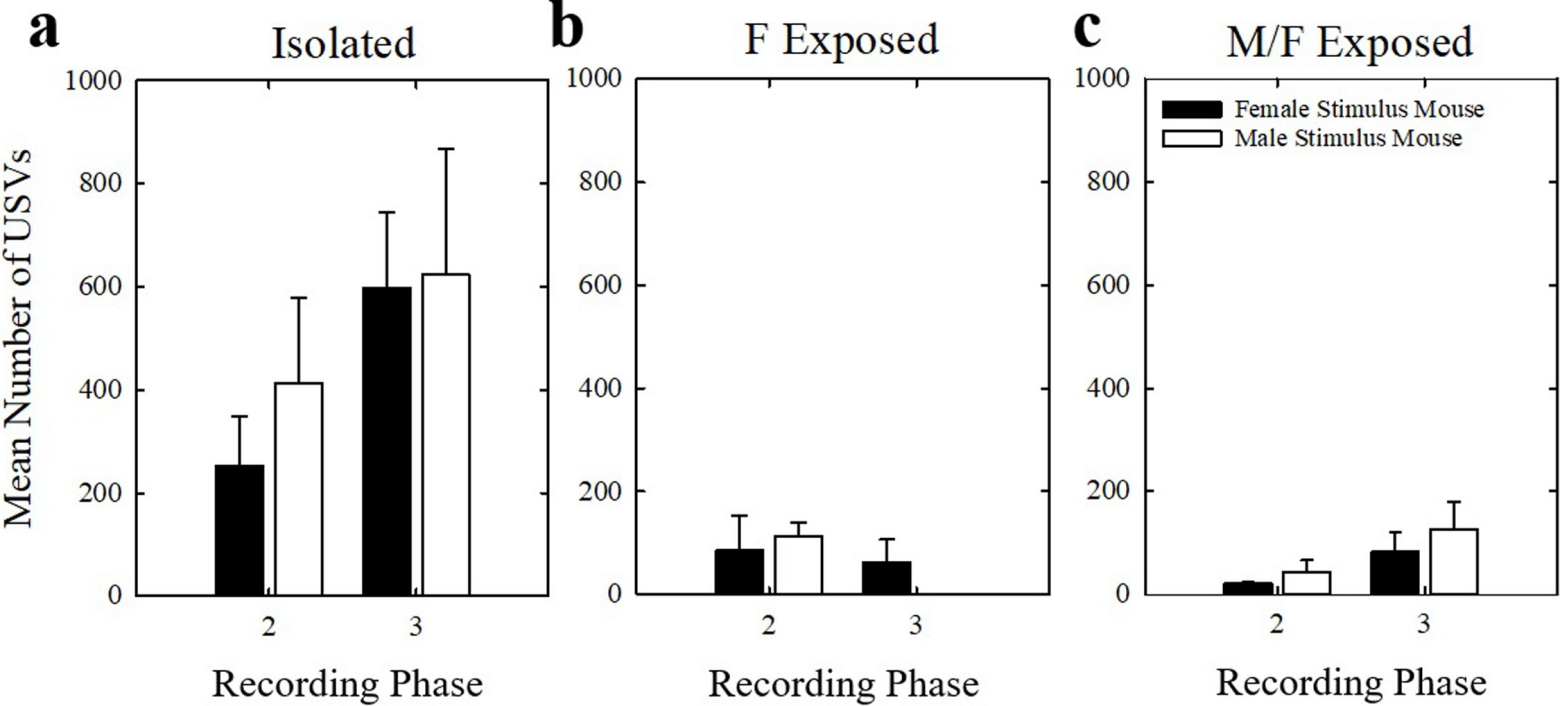
Number of USVs within housing conditions. Number of USVs produced by (a) Isolated, (b) F Exposed, and (c) M/F Exposed female mice to female stimulus mice (black bars) and male stimulus mice (white bars) in phases 2 and 3. Bars represent means and error bars represent SEM.

**Fig 6:**
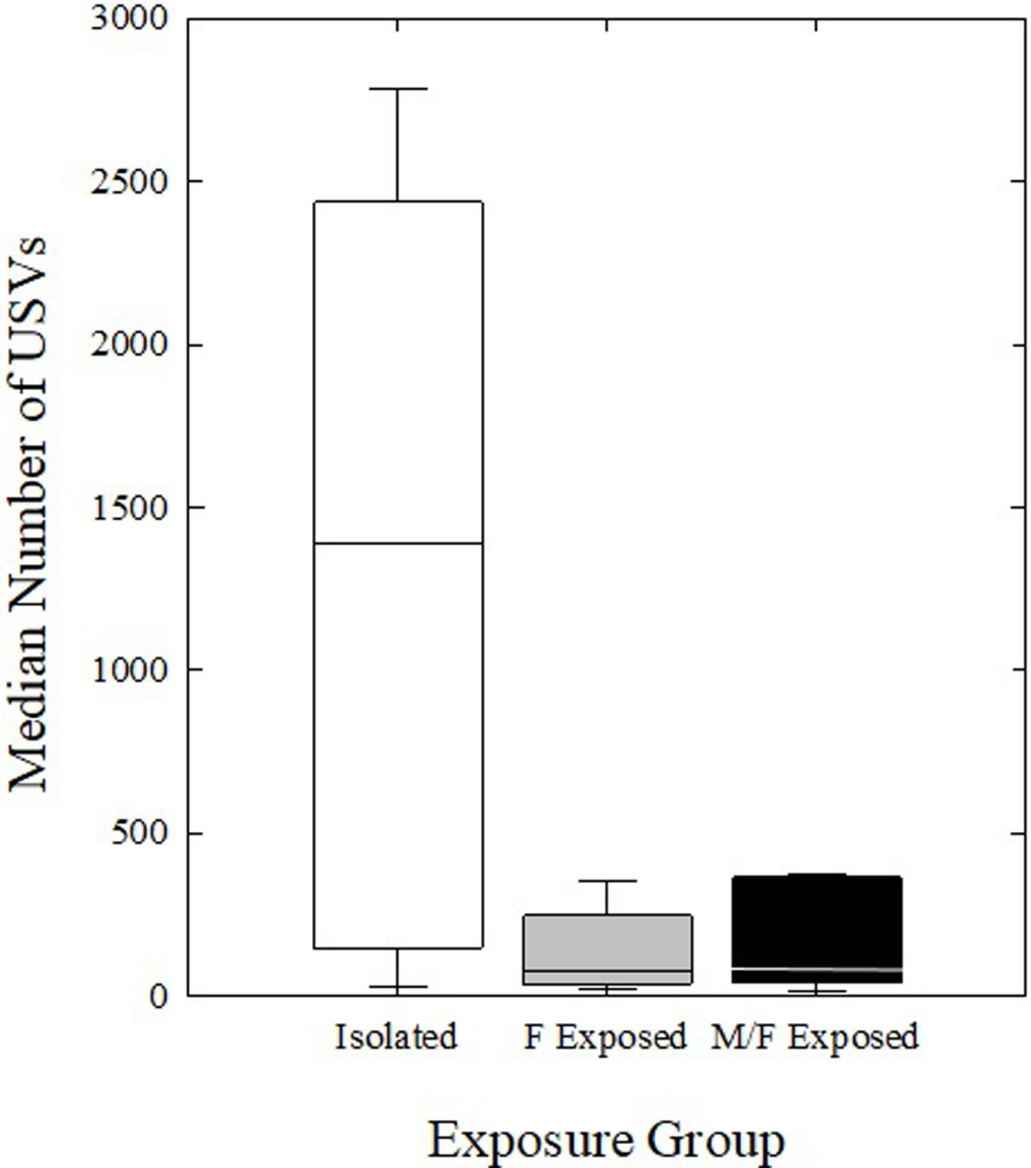
Number of USVs between housing conditions. Number of USVs produced by Isolated mice (white bars), F Exposed mice (gray bars), and M/F Exposed mice (black bars) in all four recording sessions, averaged across phases 2 and 3. Bars represent upper and lower quartile range of number of USVs, horizontal lines represent the medians.

One-way ANOVAs revealed no significant differences within housing groups between phases 2 and 3 (recording sessions 3 & 4 vs. 5 & 6) to either stimulus mouse sex in isolated, F Exposed, or M/F Exposed mice (*p* > 0.025, power < 0.45 for all comparisons) (Fig 5). USVs from phases 2 and 3 were then examined between housing groups using the mean of all four recording sessions for each mouse. There were no significant differences in the median number of USVs produced by the female mice in the three social housing groups (*H* = 5.815, df = 2, *p* = 0.055) (Fig 6).

**Fig 7:**
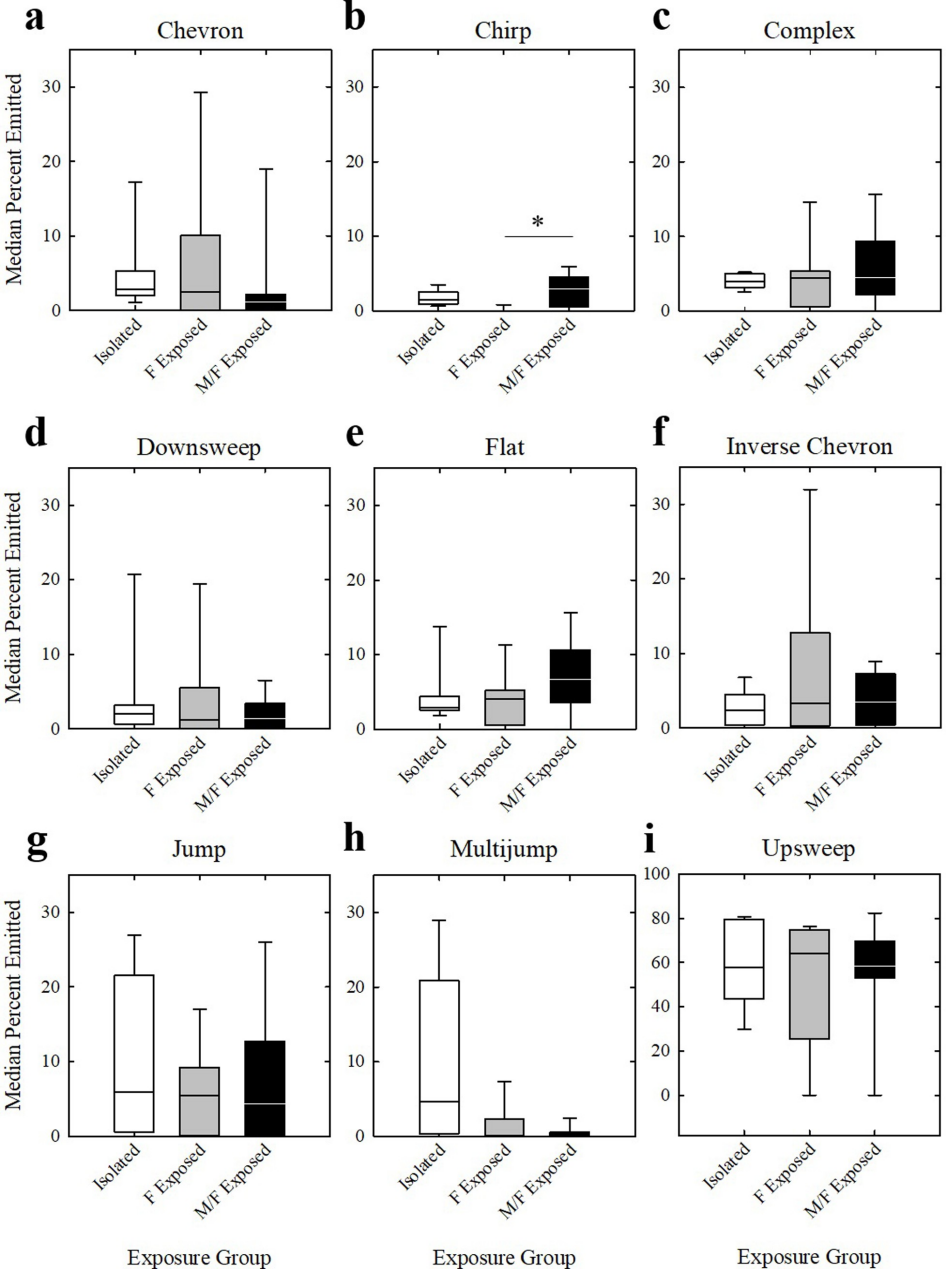
Proportion of USVs. The proportion of USVs produced by each social exposure group (Isolated = white bars, F Exposed = gray bars, and M/F Exposed = black bars) for (a) chevron, (b) chirp, (c) complex, (d) downsweep, (e) flat, (f) inverse chevron, (g) jump, (h) multijump, and (i) upsweep USV categories. Bars represent upper and lower quartile range of proportion of each USV, horizontal line represents median. * *p* < 0.025.

Proportions of USVs were first investigated within each housing group independently using Friedman tests. No significant differences emerged between recording phases for any of the three housing groups for any category (*p* > 0.025). Next, we investigated the mean proportion of USVs produced by each mouse across all recording sessions and compared the three housing conditions. Mice in the three exposure groups did not emit a significantly different proportion of any USV category (*p* > 0.025) except chirps (*H* = 11.812, *df* = 2, *p* = 0.003) (Fig 7). Dunn’s post hoc analyses revealed that F Exposed mice emitted significantly fewer chirp USVs than M/F Exposed mice (*Q* = 3.111, *p* = 0.006). All other pairwise comparisons were not significant (*p* > 0.025).

**Fig 8:**
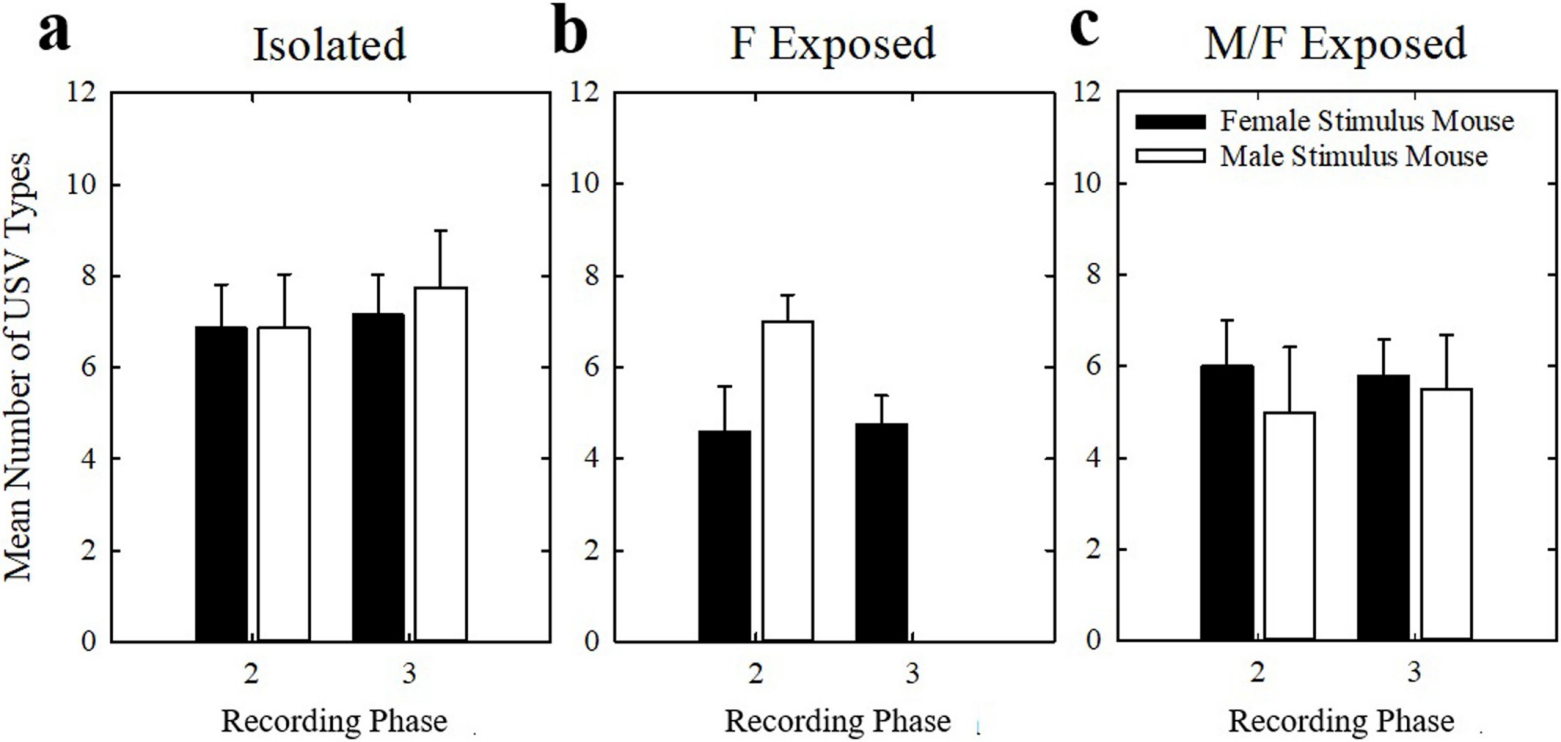
Variety of USVs produced within housing conditions. Mean number of USV types produced by (a) Isolated, (b) F Exposed, and (c) M/F Exposed mice to female stimulus mice (filled bars) and male stimulus mice (open bars) in each recording session in phases 2 and 3. Bars represent mean number of USVs emitted in a session and error bars represent SEM.

**Fig 9:**
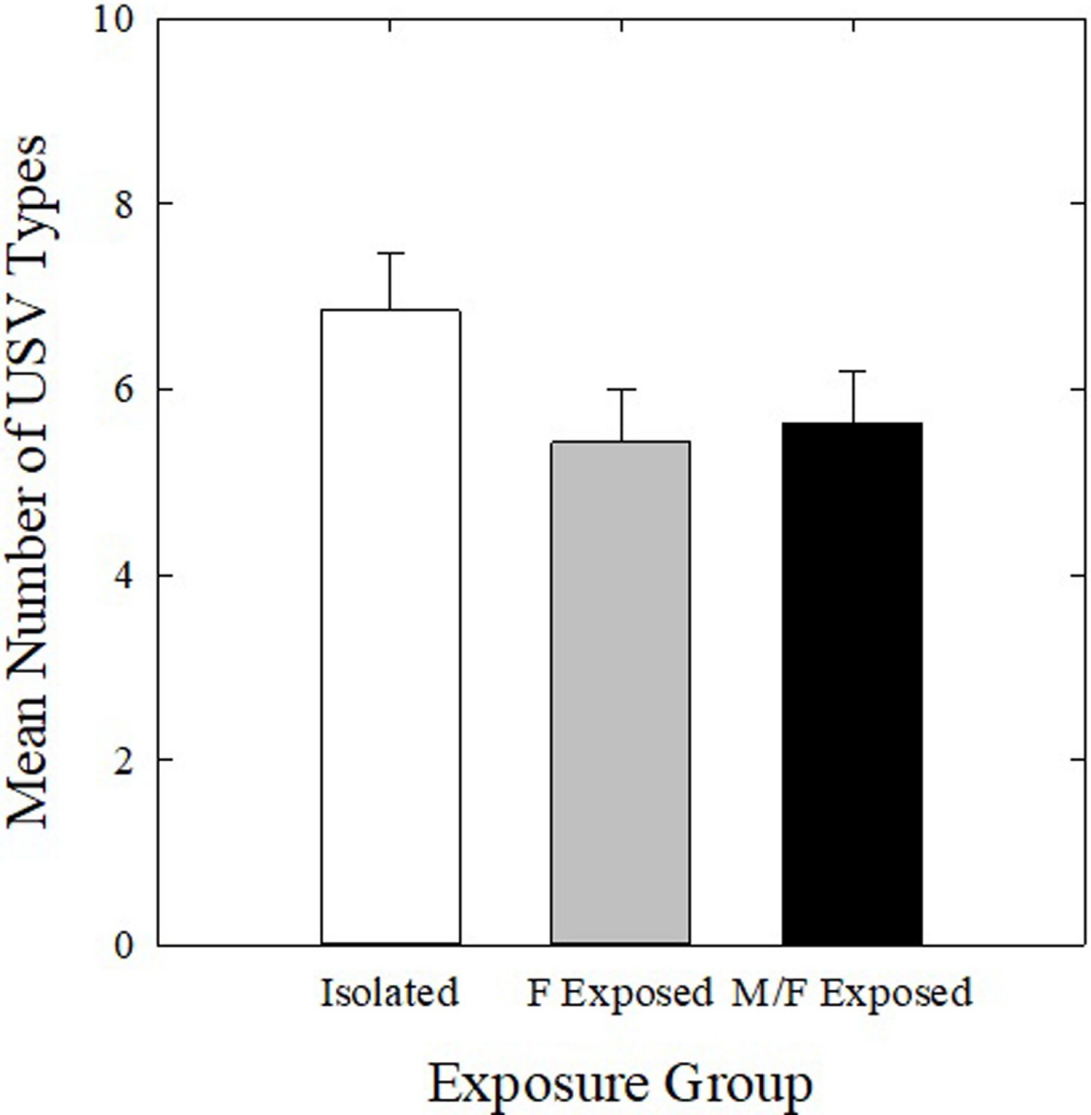
Variety of USVs produced between housing conditions. Mean number of USV types produced by Isolated (white bars), F Exposed (gray bars), and M/F Exposed mice (black bars) in all four recording sessions in phases 2 and 3. Bars represent mean number of USVs emitted in all sessions and error bars represent SEM.

The number of USV types emitted in the two recording phases within each housing condition separately was examined. There were no significant differences in mean number of USV types emitted for any housing group (*p* > 0.025; power < 0.20 for all comparisons) (Fig 8). Mean number of USV types emitted was also examined between housing conditions. There was no significant effect of housing condition on mean number of USV types emitted (*p* > 0.025; power = 0.142) (Fig 9).

**Fig 10:**
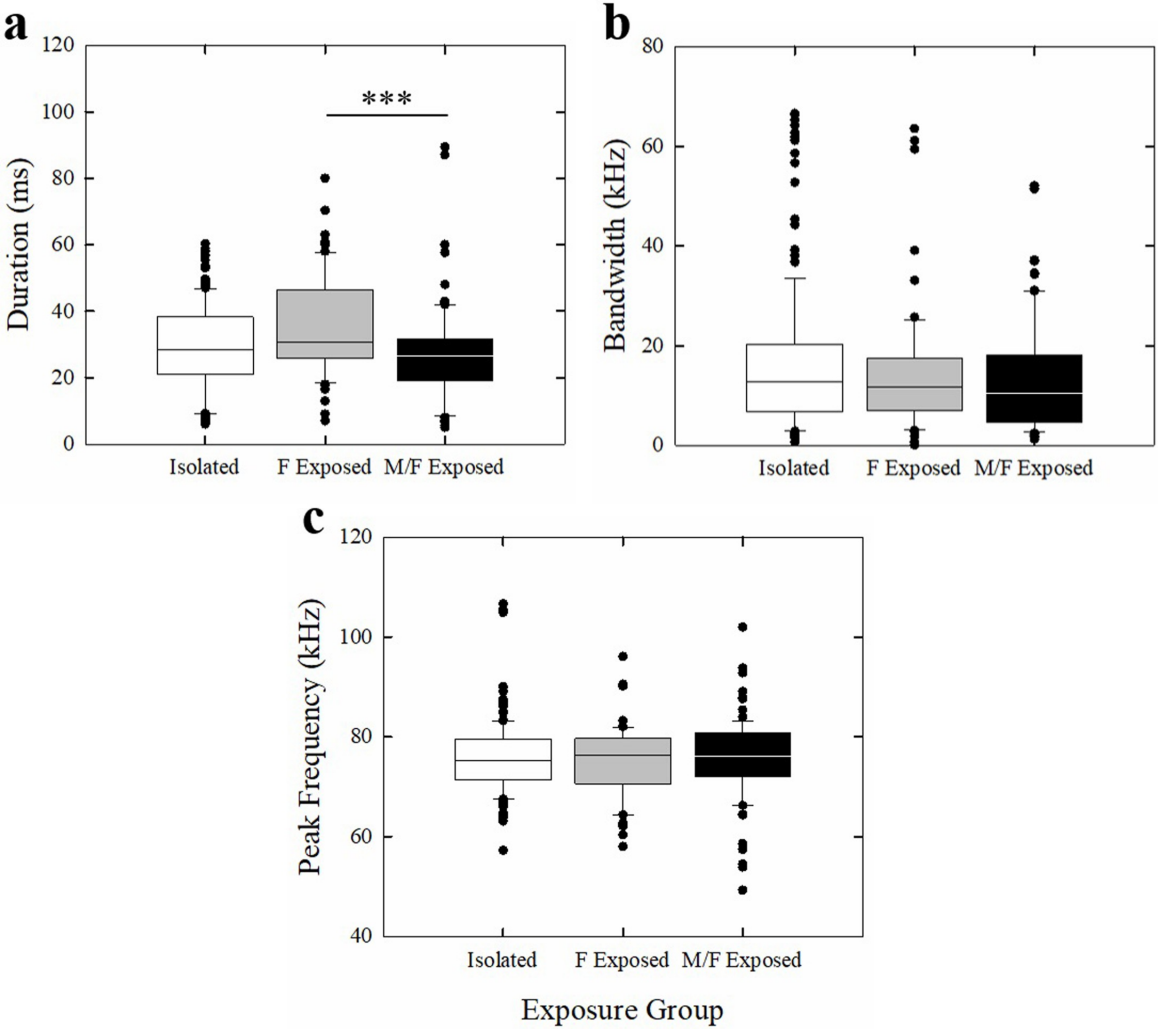
Features of USVs produced by mice in all three housing conditions. (a) Duration, (b) bandwidth, and (c) peak frequency of USVs from all categories emitted by Isolated (white bars), F Exposed (gray bars), and M/F Expose (black bars) in all recording sessions. Bars represent upper and lower quartile ranges of each feature, horizontal line represents median, circle symbols represent outliers. *** *p* < 0.001.

The features of each USV (duration, bandwidth, and peak frequency) were analyzed for all USV types across social exposure groups. There was a significant effect of housing group on duration (*H* = 12.330, *df* = 2, *p* = 0.002). Dunn’s post hoc analyses revealed there was a significant difference between the F Exposed and M/F Exposed groups, with F Exposed mice emitting significantly longer USVs than M/F Exposed mice (*Q* = 3.502, *p* = 0.001) (Fig 10A). No other comparisons were significant (*p* > 0.05). There was no significant effect of housing on bandwidth or peak frequency (*p* > 0.05) (Fig 10B and 10C).

In sum, social isolation did not lead to the production of a significantly different number of USVs or USV types. Additional social experience with males did not produce differences in vocal behavior between the F Exposed and the M/F Exposed groups. Social experience also did not lead to significant differences in the peak frequencies, durations, or bandwidths of almost all categories of USVs.

## Discussion

The goal of this study was to investigate whether social experience influenced USV production in female CBA/CaJ mice in diestrus. Previous experiments examining the influence of social experience on vocal behavior in mice were conducted exclusively in males and presented conflicting findings. Social experience was a critical component for production of USVs to olfactory signals by males [33–34, 39]. Conversely, Keesom and colleagues [21] found that social experience was not necessary for male mice to emit USVs during social interaction with another male. Keesom’s [21] chronically socially isolated male mice emitted more USVs than socially experienced mice during interactions with same-sex conspecifics. The present experiment illustrated that social experience does not play a role in USV production by females in diestrus, although our results are somewhat limited by the small sample size used. Recording mice in the diestrus phase could have had an effect on USV production, as this is a sexually non-receptive phase. It is possible that vocal behavior by isolated versus experienced females in estrus would differ since the former may be attempting to communicate about reproductive status. Thus, the influence of social experience on vocal behavior in female mice cannot be totally discounted until these conditions can be addressed.

Interestingly, the type of social experience largely did not have an effect on vocalization production by female mice. The only difference found between the USVs emitted by the mice in the F Exposed and M/F Exposed groups was that F Exposed mice produced USVs with longer durations. Keesom and colleagues [21] found that isolated male mice produce longer duration USVs than socially housed males, differing from the current results in female mice. M/F Exposed mice did not change their USV production significantly between phase 2, when these mice only had experience with females, and phase 3, when mice had experience with both males and females. It is possible that having more direct social experience with males, such as mating, would exert an effect on USV production. Nonsexual social experience with males, however, did not influence vocal behavior in female mice.

In male mice, USV production changes throughout development, from isolation calls emitted by pups to mating-induced and social USVs produced by adults. Grimsley et al. [6] investigated the developmental trajectory of male USV production. The authors found various changes occurred in USV production throughout development, including differences in frequency, duration, and temporal characteristics. Mice were also found to increase the complexity of their USV bouts in adulthood compared to those of juveniles, as shown by less repetition of syllables throughout the bout. This suggests that development is a critical component to adult USV production. Social isolation during this period could produce the differences observed by Keesom and colleagues [21] between the isolated and socially housed male mice; however, social isolation did not have an influence in the current experiment. Maturation of vocalization production has also been observed in other species, such as marmosets [40]. It is unclear if social experience during development followed by social isolation in adulthood would lead to similar changes observed in this experiment, or if differences emerged as a result of isolation during the maturation of vocal production.

The female mice in the current experiment did not produce USVs with significantly different features across different levels of social experience. Mice that did not have social interactions with either sex as an adult still produced typical USVs. These findings in female mice disagree with those of Keesom and colleagues [21], who found that socially isolated male mice produced USVs with longer durations than socially experienced mice. This difference could be the result of sex, strain, or other methodological differences between studies. Mice used in [21] were male CBA/J mice and were recorded in dyads and were therefore allowed to physically interact. The present experiment recorded from mice in isolation, so it is possible recording from two interacting mice would lead to different results. Strain-specific USVs do not require acoustic input for proper production; deaf male mice produce similar vocalizations as mice with normal hearing [8]. The present experiment illustrates that prior social interactions are also not necessary for the production of normal USVs in female CBA/CaJ mice.

It is clear from this experiment that the contribution of female mice to vocal exchanges during courtship can no longer be overlooked. Female mice in the current experiment emitted a large number of USVs to both male and female stimulus mice. The contribution of female mice’s USVs in mixed-sex dyads has been largely ignored since Whitney et al. [9] found that females do not vocalize to anesthetized mice. Additionally, Chabout et al. [17] discovered that USVs recorded from a dyad did not differ from those recorded from a male and an anesthetized female, suggesting female mice are not contributing to recorded vocalizations in pairs. The present experiment, however, demonstrates that female mice do emit USVs to both male and female mice. These results agree with those of Neunuebel et al. [10] and Heckman et al. [11], both of whom found that female mice contribute to USV bouts recorded from male-female pairs.

## Conclusions

From the results of the current experiment, social isolation does not have an effect on the USVs produced by female CBA/CaJ mice. It is possible, however, that social experience was not found to have a significant effect due to the small sample size and single phase of the estrous cycle used in this experiment. Long-term social isolation led to a nonsignificant increase in the number of USVs that females produced compared to mice that had been housed with other mice. Female mice, therefore, do not need prior social interaction with conspecifics to produce USVs to conspecifics, in contrast to what has been found in female-naïve male mice [33]. Isolated females produced a similar number of USV types within a recording session as socially experienced mice. Features of USVs largely did not differ significantly across groups, although duration did differ between F Exposed and M/F Exposed mice. This suggests mice do not need prior social contact to produce normal, strain-specific USVs. Finally, social experience with male mice did not change the vocal behavior of mice who had been socially housed with females. It is still unclear if there is a sensitive period of social experience in mice, or if social isolation exerts its effects mainly during development.

## Supporting information

S1 File The raw experimental results for individuals across all conditions. (XLSX)
